# Investigating the molecular mechanisms of Fuzheng Yiliu Shenji prescription in SH-SY5Y neuroblastoma cells

**DOI:** 10.3389/fonc.2024.1447666

**Published:** 2024-09-10

**Authors:** Xueying Zhu, Yinchu Si, Cong Gai, Zhong Li

**Affiliations:** ^1^ Department of Anatomy, School of Traditional Chinese Medicine, Beijing University of Chinese Medicine, Beijing, China; ^2^ Department of Oncology, Dongzhimen Hospital, Beijing University of Chinese Medicine, Beijing, China

**Keywords:** Fuzheng Yiliu Shenji Prescription, Neuroblastoma, SH-SY5Y, Network pharmacology, PI3K-Akt signaling pathway

## Abstract

**Background:**

Neuroblastoma is the most common extracranial solid tumor in childhood. Fuzheng Yiliu Shenji Prescription (FYSP) has shown potential in treating malignant pediatric tumors in clinical settings. This study aims to explore the molecular mechanisms behind its effects, specifically in the context of neuroblastoma cell lines.

**Objective:**

To elucidate the active compounds in FYSP and their mechanisms of action in inhibiting neuroblastoma cell viability, inducing apoptosis, and affecting the cell cycle in SH-SY5Y cells through network pharmacology and empirical validation.

**Materials and methods:**

We identified the major compounds in FYSP and their predicted targets, constructing a protein-protein interaction (PPI) network and performing GO and KEGG pathway analyses. The effects of FYSP were empirically validated through assays on cell viability, cell cycle, apoptosis, and protein expression in SH-SY5Y cells.

**Results:**

The study identified 172 active chemical components in FYSP, with 188 common targets related to neuroblastoma. Network analysis highlighted the PI3K-Akt pathway as a significant target. Experimental validation in SH-SY5Y cells confirmed that FYSP could inhibit cell viability, induce G2/M cell cycle arrest, and promote apoptosis through modulation of the PI3K-Akt pathway, specifically upregulating caspase-3 and downregulating Bcl-2/Bax expression.

**Conclusion:**

The study elucidates the molecular basis of FYSP’s effects on neuroblastoma cells *in vitro*, demonstrating its ability to modulate key pathways involved in cell cycle and apoptosis. While these findings suggest a potential therapeutic role for FYSP, they are limited to *in vitro* observations, and further research, including *in vivo* studies, is necessary to explore its clinical applicability.

## Highlights

- Qi (气) Definition: Qi represents the vital life force or energy that circulates through the body, essential for sustaining life and health. It is a fundamental concept in TCM, believed to influence the functional activities of the body. Disruptions or imbalances in Qi are thought to lead to disease.
- Zheng Jia (癥瘕) Definition: Translated as “solid masses,” this term refers to palpable tumors in TCM, indicative of a solid nature of malignancy. It is used to describe the tangible, solid aspects of tumors in pediatric oncology within TCM diagnostics.
- Ji Ju (积聚) Definition: This term means “accumulation of toxins” and describes pathological states where toxins accumulate in the body, contributing to tumor growth and exacerbating the disease process in TCM theory.
- Delicate Organs and Insufficient Form and Qi (脏腑娇嫩, 形气未充) Definition: A description used in TCM to illustrate a condition where a child’s organs are considered inherently weak or fragile, with a lack of sufficient energy and physical strength, making them more susceptible to illness.
- Clear and Spirited Organ Qi (脏气清灵) Definition: This phrase describes the inherent vitality and resilience found in children’s organ systems, according to TCM. It implies that despite their vulnerability, children have a natural propensity for recovery and healing.
- FYSP (扶正抑瘤神机方) Definition: An acronym for Fuzheng Yiliu Shenji Prescription, a TCM formula used widely in oncology, particularly for treating malignant pediatric tumors. The formula is designed to strengthen the body’s righteous energy while suppressing cancerous growths.
- Fetal Toxins (胎毒) Definition: A concept in TCM that attributes certain pathological conditions in children to toxins accumulated from the mother, often related to the mother’s diet or environment during pregnancy. These toxins are believed to affect the child’s health post-birth.

## 1 Introduction

Malignant tumors are one of the leading causes of death in children worldwide (1). The World Health Organization (WHO) defines pediatric malignancies as cancers occurring in children and adolescents under the age of 15, which include leukemias, brain tumors, lymphomas, and solid tumors such as neuroblastoma and Wilms tumor (2). The Global Burden of Childhood Cancer Study in 2019 identified pediatric cancer as the ninth major burden of disease for children worldwide, with China ranking second globally (3). The report highlighted that in low- and middle-income countries, the recovery rate for childhood malignancies is less than 30% (3). Despite improvements in economic and medical standards that have continuously raised the treatment and cure rates for pediatric malignancies, the cancer survival time for affected children has progressively extended. Traditional Chinese Medicine (TCM) has gained attention in the oncology field due to its efficacy, minimal adverse reactions, and multi-pathway synergistic interventions, with its advantages increasingly being recognized (4, 5).

Neuroblastoma, the most common extracranial solid tumor in childhood, presents unique challenges due to its diverse staging system which significantly impacts prognosis and treatment strategies. Accounting for 8% of pediatric malignancies, neuroblastoma predominantly affects infants, with a median age of diagnosis at 18 months and 90% of cases occurring in children under five (6, 7). Crucially, neuroblastoma is classified into five stages (1, 2, 3, 4, and 4S). The advanced stages 3 and 4 are particularly notorious for being high-risk and chemotherapy-resistant, often characterized by MYCN amplification, mutations in ALK and ATRX, and genomic rearrangements in TERT genes. These genetic markers contribute to the disease’s aggressive behavior and are pivotal for targeting therapeutic interventions (8). This staging and genetic profiling underscore the critical need for targeted research and therapeutic strategies.

The phosphatidylinositol 3-kinase (PI3K)/Protein Kinase B (AKT) pathway is an essential pro-survival signaling pathway activated in most neuroblastoma cases (9). Neuroblastoma is characterized by aberrant receptor tyrosine kinase (RTK) activity, primarily due to mutations or overexpression of growth factor receptors or their ligands. This is associated with increased activation of the PI3K/Akt/mTOR pathway (10). In neuroblastoma, the PI3/Akt/mTOR pathway has been found to affect multiple pathways/proteins to enhance the neuroblastoma phenotype, such as stabilizing MYCN, which in turn facilitates processes associated with malignancy like proliferation, angiogenesis, and metabolic reprogramming (11, 12). A study by Chesler et al. demonstrated that inhibition of the PI3/Akt/mTOR pathway leads to reduced N-Myc protein levels in neuroblastoma (12).

Professor Li Zhong, a renowned expert in TCM oncology, has extensively utilized the Fuzheng Yiliu Shenji Prescription (FYSP) in the management of malignant pediatric tumors. In TCM practice, these tumors are classified into categories such as ‘Zheng Jia’ (solid masses) and ‘Ji Ju’ (accumulated toxins), which describe specific pathological states recognized in pediatric patients. ‘Zheng Jia’ refers to solid, palpable tumors, indicative of malignant growth, while ‘Ji Ju’ denotes toxin accumulation in the body, influencing tumor growth and behavior. This categorization reflects traditional views on pediatric physiology, characterized by ‘delicate organs and insufficient form and qi’—a term implying vulnerability to illness due to weak bodily structure and energy—and ‘clear and spirited organ qi, prone to recovery’, suggesting a natural propensity for healing and vitality in children. These traditional diagnostic categories, deeply rooted in TCM, provide a framework for tailored treatment strategies in pediatric oncology (13). He emphasizes the primary importance of reinforcing health to strengthen the correct qi. Additionally, combining the concept of “fetal toxins” as a causative factor, he relates it to “maternal consumption of toxic substances”, leading to “fetal exposure and subsequent disease emergence post-birth”. Therefore, beyond reinforcing the correct qi, detoxifying and resolving masses are also necessary to suppress tumor growth. Based on this understanding of the pathogenesis of malignant pediatric tumors, Professor Li formulated the basic prescription for treating pediatric malignancies—FYSP, which includes ingredients such as turtle shell, astragalus, roasted astragalus, dark plum, angelica, prince ginseng, cornelian cherry, chicken gizzard lining, dragon bone, gold thread cucumber, licorice, Zhejiang fritillary, and crane herb. To explore the potential anti-tumor mechanisms of FYSP, this study employs network pharmacology and related experimental methods to deeply investigate its mechanisms of action.

## 2 Materials and methods

### 2.1 Screening of active components and construction of the “drug-active component-target” network

Active components and their target proteins from the plants used in FYSP were collected from the TCM Systems Pharmacology database (TCMSP, https://www.tcmsp-e.com/#/database). Bioactive compounds were selected based on an oral bioavailability (OB) value >30% and drug-likeness (DL) value >0.18. Active components in animal and mineral medicines were identified using TCMID (http://www.megabionet.org/tcmid/). Chemical structures were retrieved from NCBI PubChem (http://pubchem.ncbi.nlm.nih.gov/). Potential targets of these compounds were predicted using the SwissTargetPrediction (http://www.swisstargetprediction.ch/) and PharmMapper databases (http://www.lilab-ecust.cn/pharm-mapper/). The corresponding genes were searched in the UniProt database (https://www.uniprot.org/). Data were imported into Cytoscape 3.7.2 software to construct the “Drug-Active Component-Target” network.

### 2.2 Collection of targets related to pediatric malignant tumors

Target search terms such as “Right adrenal neuroblastoma, left adrenal neuroblastoma, adrenal neuroblastoma, neuroblastoma, mediastinal neuroblastoma, retroperitoneal neuroblastoma tumor” were used in Genecards (https://www.genecards.org/) and OMIM databases (https://www.omim.org/) to find relevant targets.

### 2.3 Construction of the “FYSP-target-disease” network

To elucidate the interactions between active components of FYSP and disease targets, a “Drug Target” and “Disease Target” Venn diagram was created using Venny 2.1.0. The intersection of these two groups of targets identified common targets, which were imported into Cytoscape 3.7.2 software to build a “FYSP-Target-Disease” visualization network.

### 2.4 Construction of PPI network

The preliminary protein interaction network was formed by merging the targets related to FYSP and pediatric malignant tumors using Cytoscape 3.7.2 software. This network was analyzed using BisoGenet 3.0 software. Core target genes were revealed by calculating topological parameters such as degree centrality (DC), betweenness centrality (BC), and closeness centrality (CC) using the CytoNCA plugin in Cytoscape.

### 2.5 GO and KEGG enrichment analysis

Key targets were analyzed for Gene Ontology (GO) and Kyoto Encyclopedia of Genes and Genomes (KEGG) pathway enrichment using the Metascape platform (http://metascape.org). GO enrichment analysis was performed across three categories: biological process (BP), molecular function (MF), and cellular component (CC).

### 2.6 Cell lines and animals

Human neuroblastoma (SH-SY5Y) cell lines were purchased from the National Biomedical Experimental Cell Resource Database. Cells were cultured in DMEM high glucose medium supplemented with 10% fetal bovine serum and 1% penicillin-streptomycin at 37°C in a 5% CO2 incubator. Twenty SPF-grade male SD rats, approximately (260 ± 20) g, were acclimatized for seven days under controlled conditions (20-25°C, 12-hour light/dark cycle), with unrestricted access to food and water. The experimental protocol was approved by the Animal Ethics Committee of Dongzhimen Hospital, Beijing University of Chinese Medicine (2023DZMEC-093-02), and conducted in accordance with the guidelines of the Chinese Medical Association’s Committee on Animal Research Ethics (2021-007).

### 2.7 Preparation of medicinal serum

FYSP consists of turtle shell 15g, astragalus 15g, roasted astragalus 15g, dark plum 15g, angelica 10g, prince ginseng 10g, cornelian cherry 15g, chicken gizzard lining 15g, dragon bone 10g, gold thread cucumber 6g, Zhejiang fritillary 8g, crane herb 15g, and licorice 8g. These were purchased from the herb pharmacy of Dongzhimen Hospital, Beijing University of Chinese Medicine, in granular form. The clinical dosage for patients is 18g/60kg/d = 0.3g/kg/d. The equivalent dose between humans and animals was calculated according to the surface area calculation table provided in “Methodology of Pharmacological Research in TCM”, adjusting the dosage for rats by a factor of 6.3 times the human dosage. Thus, the experimental doses for rats were set at 0.945g/kg/d for the low dose group, 1.89g/kg/d for the medium dose group, and 3.78g/kg/d for the high dose group.

Rats were randomly divided into a blank serum preparation group and a FYSP medicinal serum preparation group. The blank serum group was administered saline by gavage twice daily for seven days; the medicinal serum group was divided into low, medium, and high dose groups, receiving their respective doses by gavage twice daily for seven days. One hour after the last administration, rats were anesthetized with sodium pentobarbital via intraperitoneal injection. After successful anesthesia, skin and fascia were incised to collect blood from the abdominal aorta. The blood was then centrifuged at 1500rpm in a 4°C environment for 15 minutes. The supernatant was collected and inactivated in a 56°C water bath for 30 minutes and subsequently filtered through a 0.22 µm filter in a sterile environment. The filtered serum was stored in sterile centrifuge tubes at -80°C and thawed slowly at -20°C and then 4°C before use.

### 2.8 siRNA construction and transfection

si-PI3K was constructed by Sangon Biotech. Cells were removed from the incubator, old culture medium was discarded, and the plates were washed three times with PBS. 250µL of serum-free Opti-MEM medium was added, followed by 250µL of transfection solution dropped into the corresponding wells. The culture plate was gently shaken to ensure even distribution, then incubated in a 37°C, 5% CO2 incubator. After 4 hours, 1.5mL of complete medium was added to each well, and the cells were cultured for an additional 24 hours at 37°C with 5% CO2. The medium was changed normally after 24 hours, and cells were collected for subsequent experiments after 48 hours.

### 2.9 Cell viability assay

The effect of FYSP on SH-SY5Y cell viability was assessed using the MTT assay. Cell suspension concentration was adjusted, and 100µL was added to each well of a 96-well plate to reach a cell density of 2000 cells per well. Cells were cultured at 37°C in 5% CO2 for 4 hours until adherence, after which medicated serum medium was added. MTT was added after 0, 24, and 48 hours of culture, followed by incubation in the dark for 4 hours. The optical density (OD) was measured at 490 nm wavelength using a BioTek instrument (USA). The experiment was repeated at least three times.

### 2.10 Scratch assay to assess cell migration

After cell digestion, cells were plated in a 6-well plate and cultured overnight until the cell density was nearly confluent. A scratch was made perpendicular to a ruler line using a pipette tip. Cells were washed three times with PBS to remove detached cells, and various serum media were added. The cells were then cultured at 37°C with 5% CO2 and photographed at 0, 24, and 48 hours.

### 2.11 Cell cycle analysis

SH-SY5Y cells were placed in a 6-well plate. After exposure to medicated serum for 24 and 48 hours, cells were collected and fixed overnight with cold 70% ethanol. The next day, cells were exposed to propidium iodide (PI) for 15 minutes, and the cell cycle was analyzed using a FACS Calibur flow cytometer. The experiment was repeated three times.

### 2.12 Flow cytometry to detect cell apoptosis

Cells were cultured for 24 and 48 hours before collecting and assessing apoptosis using flow cytometry. Cells from each group were digested, and 5×10^5 cells were resuspended in 500µL 1×Binding Buffer. 5µL of Annexin V-FITC and 10µL of PI staining solution were added, followed by incubation in the dark for 20 minutes. Apoptosis was then analyzed using a FACS Calibur flow cytometer. Independent experiments were repeated three times.

### 2.13 Western blotting analysis

Primary antibodies against Bax (ab32503), Bcl-2 (ab32124), Caspase-3 (ab184787), GAPDH (ab8245), PI3K (ab302958), and AKT (ab38449) were provided by Abcam, USA. Total proteins from MGC-803 and HGC-27 cells were extracted using cell lysis buffer. After SDS-PAGE and transfer to PVDF membranes, the membranes were blocked with 5% BSA and incubated with primary antibodies overnight, followed by secondary antibodies at room temperature. The membranes were then exposed to Western LightningTM Chemiluminescence Reagent for 30 seconds and immediately placed in an exposure box. Films were exposed for 1 minute in a darkroom, then developed and fixed. Films were scanned using an Epson Perfection V39 scanner, and the brightness values of protein bands were analyzed. The method involved calculating the ratio of the brightness of each sample’s protein band to that of the corresponding GAPDH (internal control) band, to obtain normalized protein band brightness values.

### 2.14 Statistical analysis

Statistical analyses were performed using SPSS software (version 22.0, IBM Corp., Armonk, NY, USA). All experimental data are presented as mean ± standard deviation (SD). Comparisons between two groups were conducted using Student’s t-test, while multiple group comparisons were performed using one-way ANOVA followed by a *post hoc* Tukey’s test. A p-value of less than 0.05 was considered statistically significant. All experiments were repeated at least three times to ensure reproducibility.

## 3 Results

### 3.1 Exploration of potential targets for pediatric malignancies using network pharmacology based on FYSP

#### 3.1.1 Active components and target prediction of FYSP

From the databases TCMSP, TCMID, SwissTargetPrediction, PharmMapper, and literature, a total of 172 active compounds and 3589 compound targets were identified, derived from 11 medicinal components of FYSP. No active compounds or targets were identified for gold thread cucumber. The number of active compounds per medicinal herb, the number of targets, and the criteria for selection are detailed in **Table 1**.

**Table 1:** The number of potential activated compounds for each TCM and the number of targets and screening basis for the role of core Chinese herbal medicine.

Serial No.	Chinese Herb	Number of Active Compounds	Number of Targets	Selection Criteria
1	Astragalus	20	462	OB>30%, DL>0.18
2	Angelica	2	70	OB>30%, DL>0.18
3	Dark Plum	8	302	OB>30%, DL>0.18
4	Prince Ginseng	8	125	OB>30%, DL>0.18
5	Cornelian Cherry	20	130	OB>30%, DL>0.18
6	Licorice	92	1766	OB>30%, DL>0.18
7	Zhejiang Fritillary	7	70	OB>30%, DL>0.18
8	Crane Herb	5	305	OB>30%, DL>0.18
9	Turtle Shell	4	221	Probability>0
10	Chicken Gizzard Lining	4	122	Probability>0
11	Dragon Bone	2	16	Probability>0

OB indicates oral bioavailability; DL denotes drug-likeness.

Basic information about some of the active components of FYSP is presented. While we recognized a broad spectrum of active compounds, this study particularly focuses on 25 of these compounds due to their higher relevance and potential impact on the therapeutic outcomes of FYSP. This selection was based on several key criteria:

- Biological significance: Compounds directly impacting the biochemical pathways associated with the specific therapeutic outcomes of interest were prioritized.
- Evidence of activity: Compounds supported by robust pharmacological evidence demonstrating efficacy or essential activity in preclinical models were selected.
- Potential for clinical applicability: Preference was given to compounds with properties suggesting a higher potential for successful clinical translation and application.

These criteria were intended to narrow down the extensive list to those compounds most likely to yield significant insights into the pharmacological basis of FYSP’s efficacy. Basic information about the selected active components, focusing on these 25 compounds, is provided in **Table 2**, offering a detailed insight into their characteristics and the rationale for their selection.”

**Table 2:** Basic information on some active ingredients of FYSP.

Serial No.	PubChem CID	Chemical Name	OB(%)	DL	Chemical Structure Diagram	Serial No.	PubChem CID	Chemical Name	OB(%)	DL	Chemical Structure Diagram
**1**	64971	Mairin	55.38	0.78	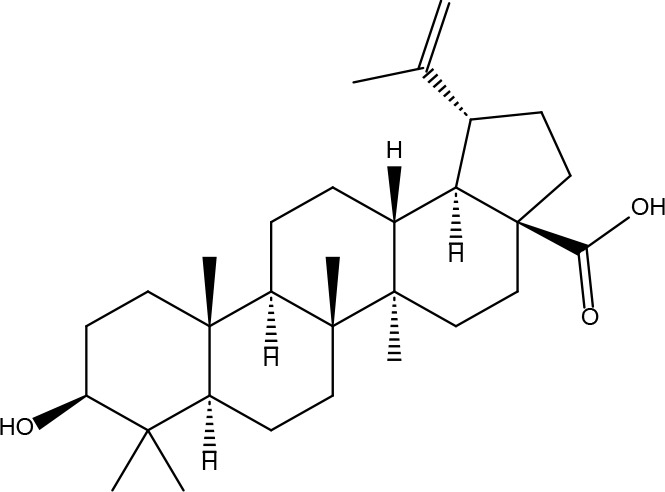	**15**	135398658	Folic Acid	68.96	0.71	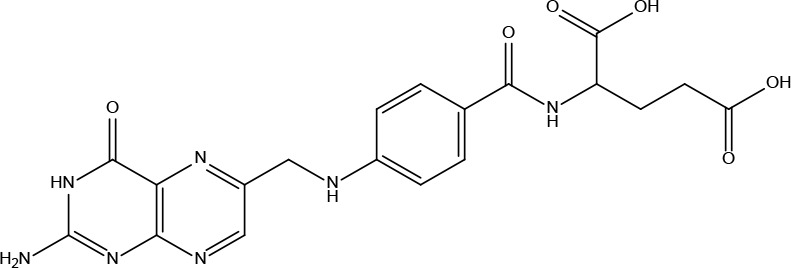
**2**	5318869	Jaranol	50.83	0.29	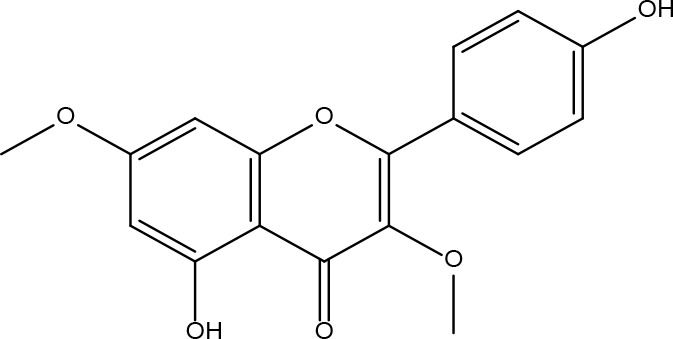	**16**	10380176	(3R)-3-(2-hydroxy-3,4-dimethoxyphenyl)chroman-7-ol	67.67	0.26	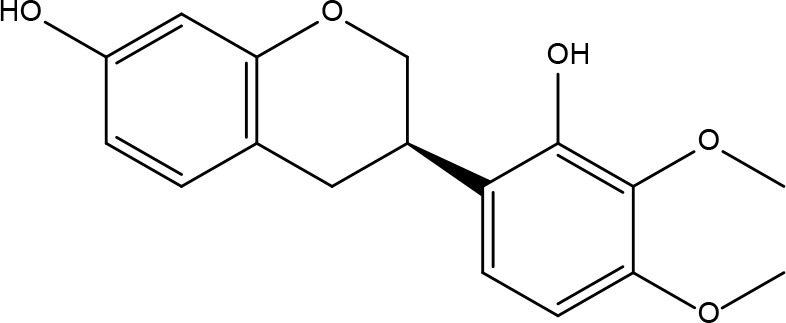
**3**	73299	Hederagenin	36.91	0.75	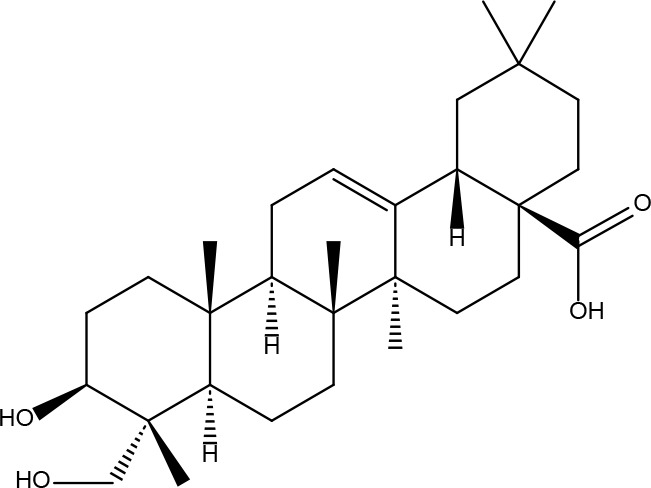	**17**	15689653	Isomucronulatol-7,2’-di-O-glucosiole	49.28	0.62	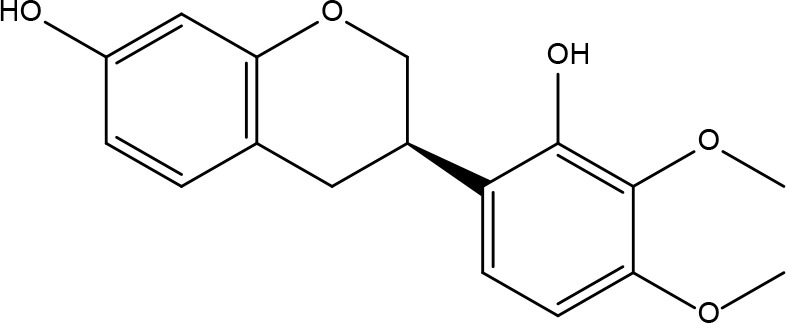
**4**	15976101	(24S)-24-Propylcholesta-5-ene-3beta-ol	36.23	0.78	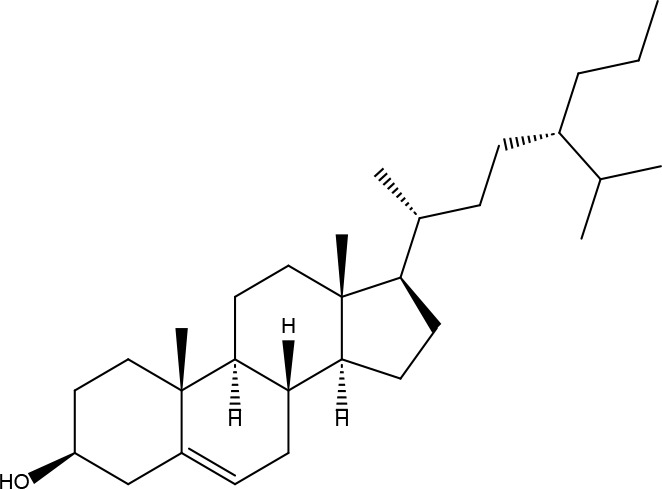	**18**	5316760	1,7-Dihydroxy-3,9-dimethoxy pterocarpene	39.05	0.48	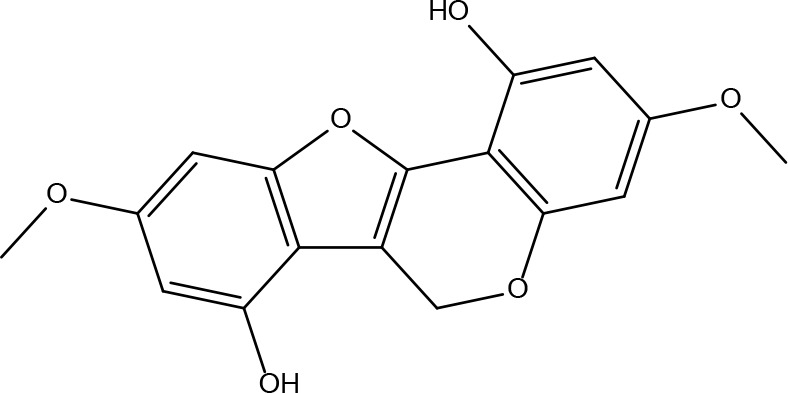
**5**	5281654	isorhamnetin	49.6	0.31	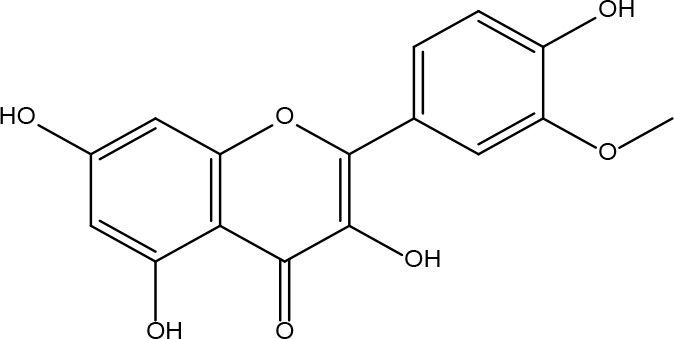	**19**	5280343	Quercetin	46.43	0.28	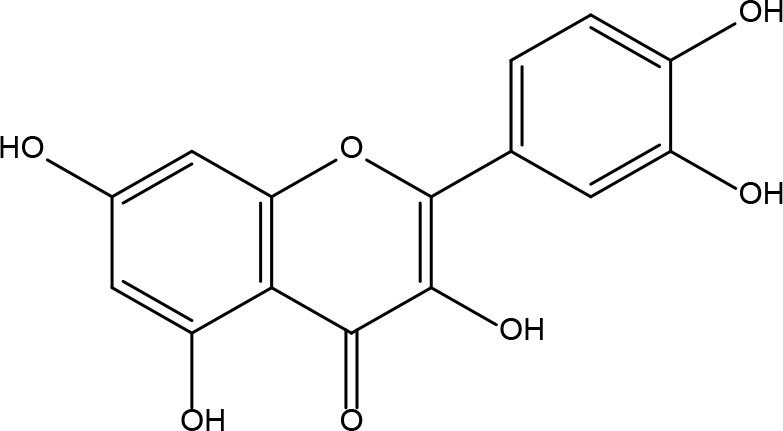
**6**	162842488	5’-hydroxyiso-muronulatol-2’,5’-di-O-glucoside	41.72	0.69	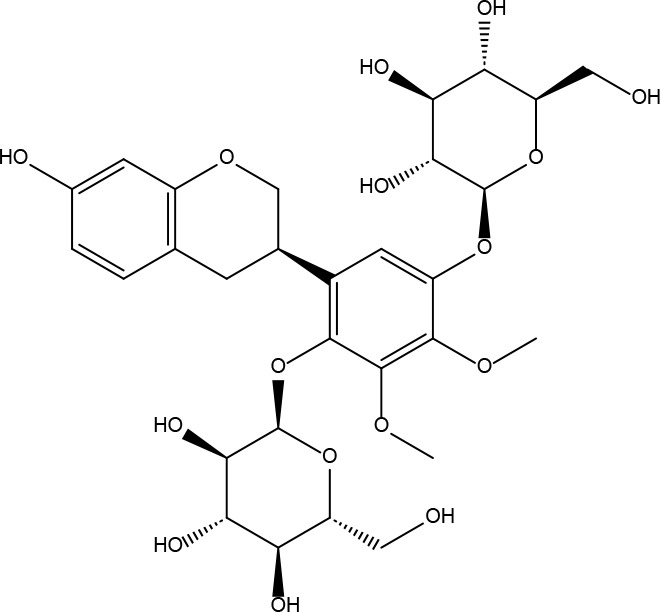	**20**	5280794	Stigmasterol	43.83	0.76	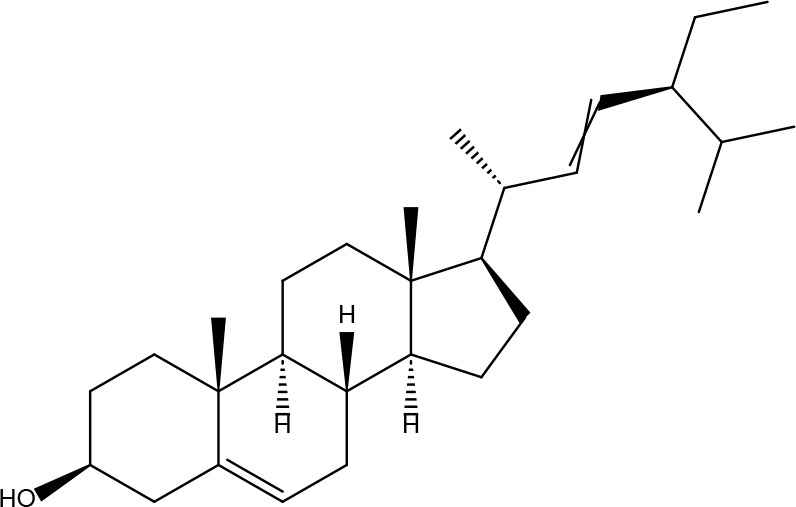
**7**	15689652	(R)-2,3-Dimethoxy-6-(7-methoxychroman-3-yl)phenol	74.69	0.3	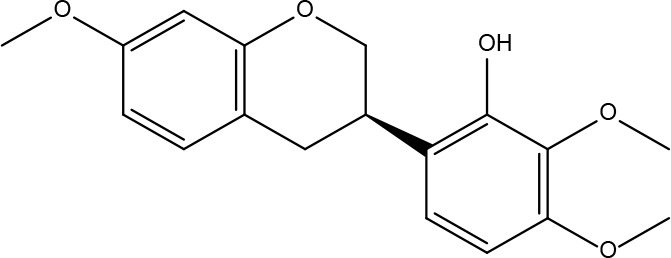	**21**	667495	(2R)-5,7-dihydroxy-2-(4-hydroxyphenyl)chroman-4-one	42.36	0.21	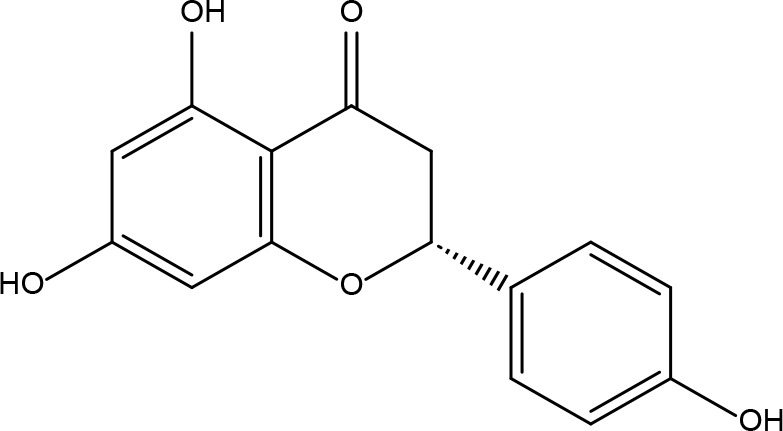
**8**	101679160	9,10-dimethoxypterocarpan-3-O-β-D-glucoside	36.74	0.92	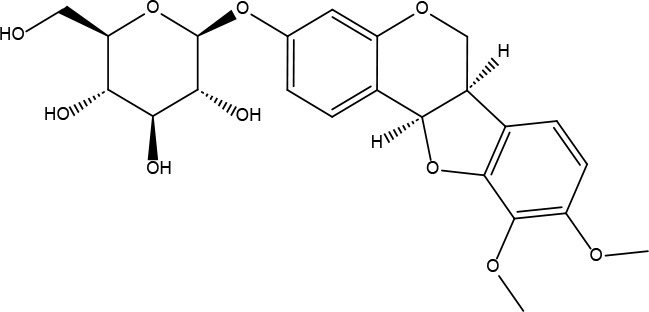	**22**	173183	campest-5-en-3beta-ol	37.58	0.71	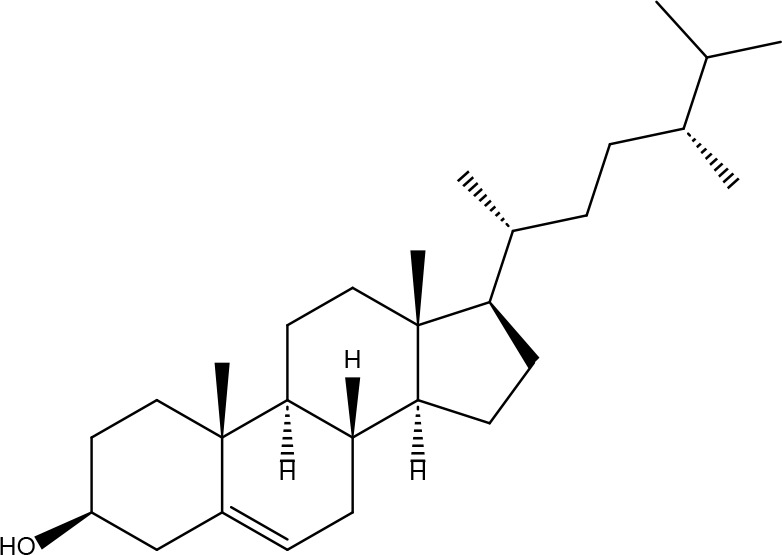
**9**	14077830	Astrapterocarpan	64.26	0.42	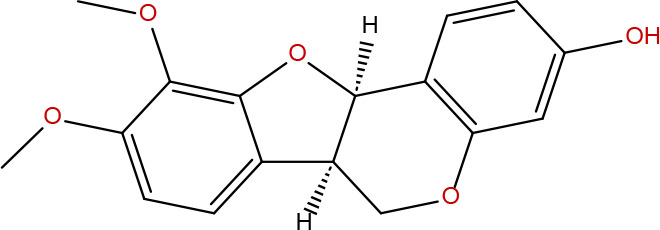	**23**	6421258	Methyl arachidonate	46.9	0.23	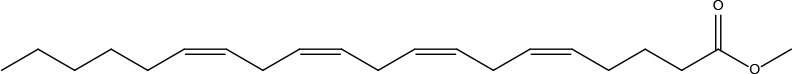
**10**	108213	Bifendate	31.1	0.67	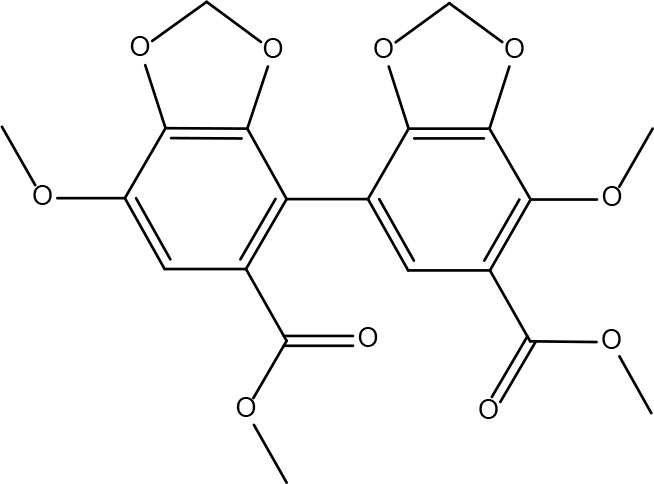	**24**	5997	Cholesterol	37.87	0.68	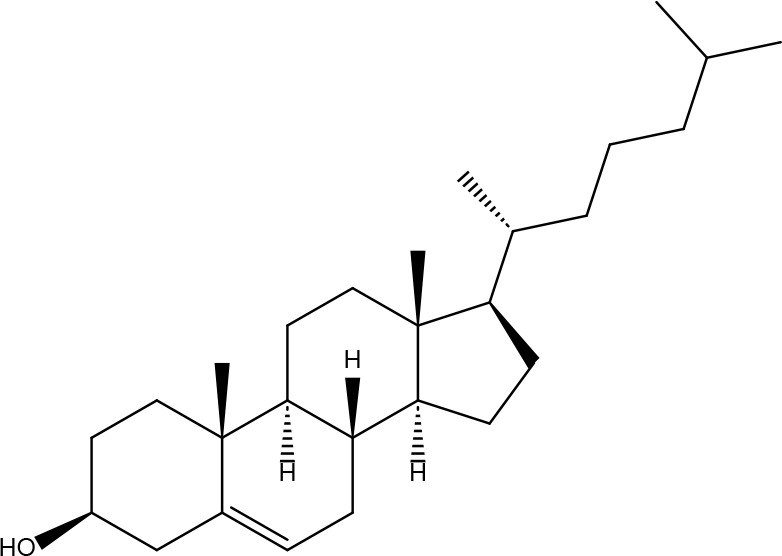
**11**	5280378	Formononetin	69.67	0.21	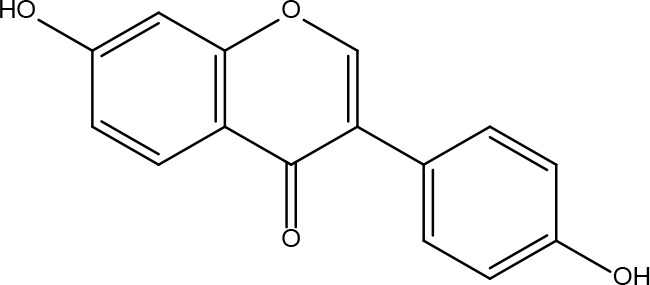	**25**	5319737	8,11-Octadecadienoic acid	55.38	0.78	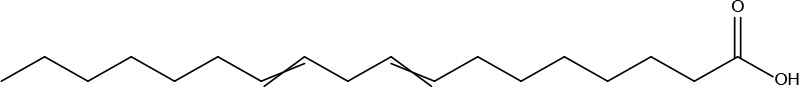
**12**	160767	Isoflavanone	109.99	0.3	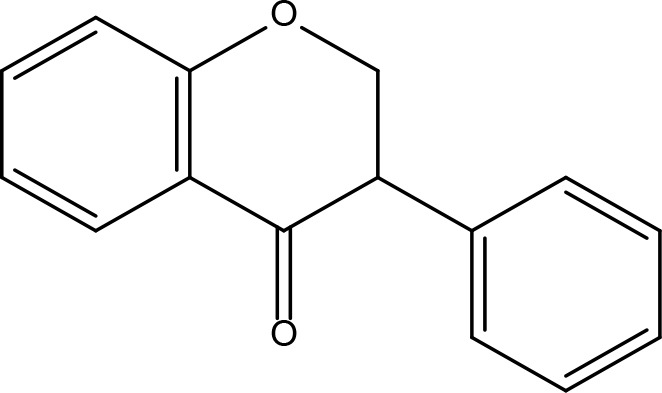	**26**	10465	Heptadecanoic acid			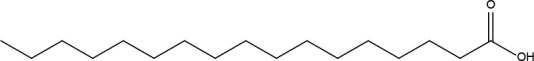
**13**	5280448	Calycosin	47.75	0.24	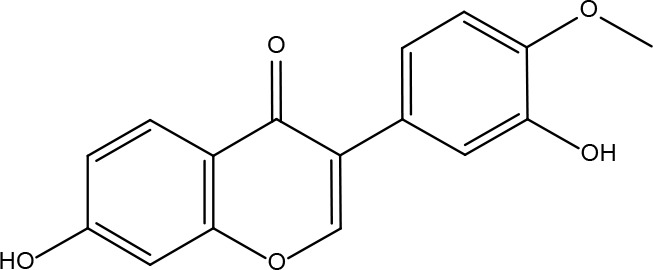	**27**	5460703	Vitamin D4			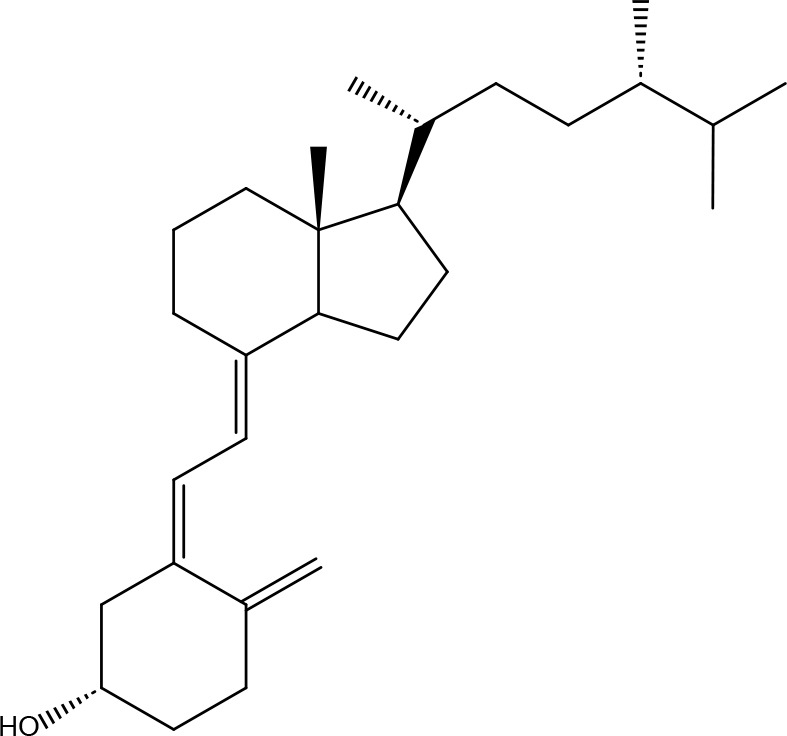
**14**	5280863	Kaempferol	41.88	0.24	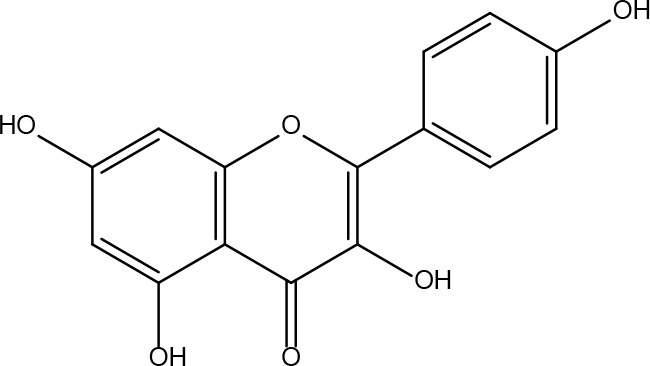	**28**	5282758	8-Octadecenoic acid			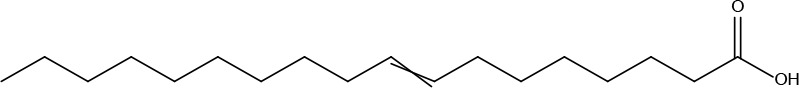

#### 3.1.2 Construction of the “drug-active component-target” network for FYSP

**Figure 1:**
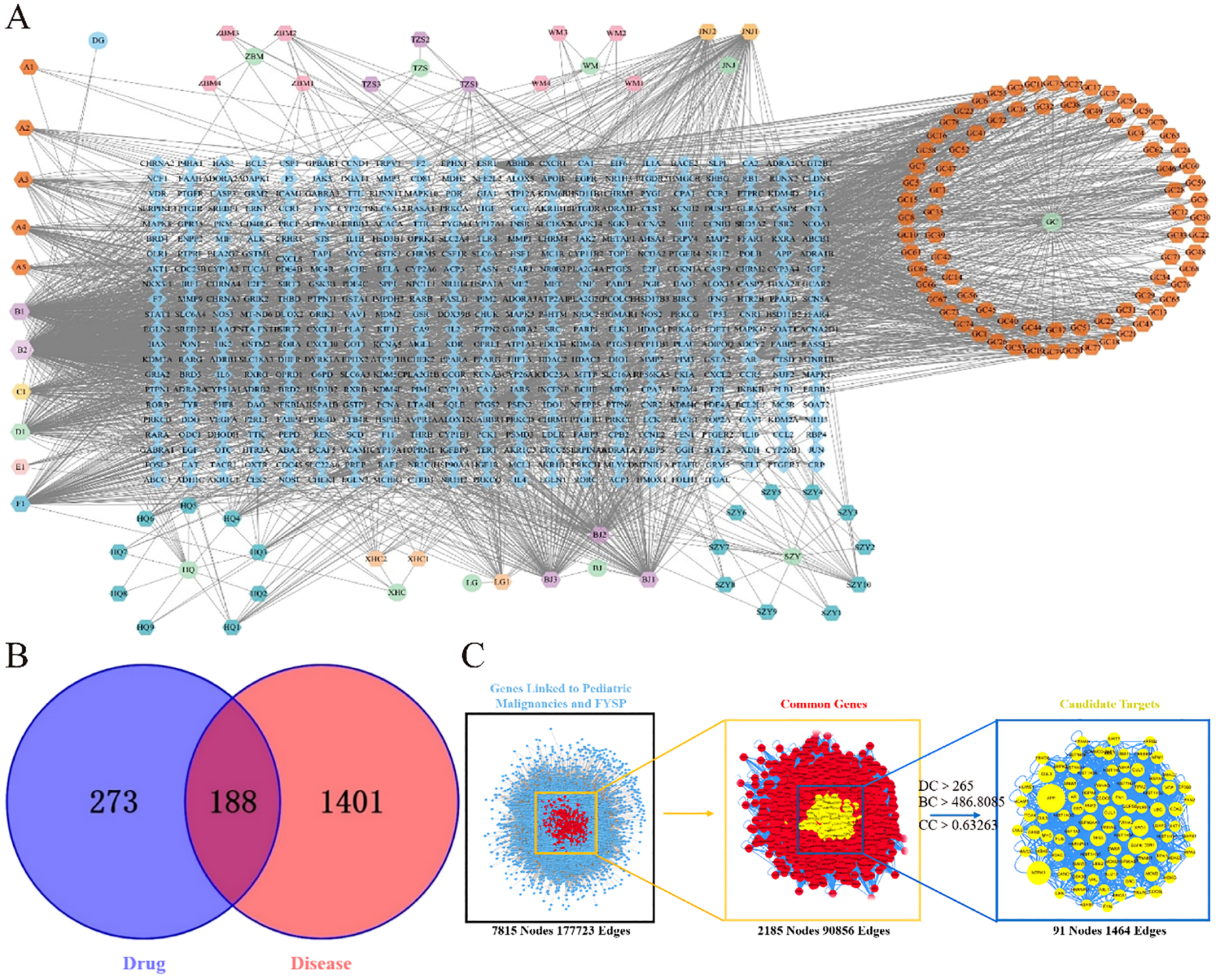
**(A)** FYSP “Drug-Active ingredients-Target” network. Circles represent drugs, hexagons represent compounds derived from the drugs, and diamonds represent the targets of the drugs in the network diagram.BJ Turtle shell, HQ Astragalus, WM Dark plum, DG Angelica, TZS Prince ginseng, WZY Cornelian cherry, JNJ Chicken gizzard lining, LG Dragon bone, ZBM Zhejiang fritillary, XHC Crane herb, GC Licorice; **(B)** Venn diagram of FYSP and pediatric tumor targets. blue circles represent drug targets, red circles represent disease targets, and the overlapping area denotes potential therapeutic targets; **(C)** Topological network of FYSP for the treatment of pediatric tumor.

To elucidate the relationships among drugs, components, targets, and pathways more clearly, a network diagram was constructed. The drugs, targets, and pathways were imported into Cytoscape 3.7.2 to build a network diagram that includes “Drug-Active Component-Target-Pathway”. The “Drug-Active Component-Target” network for FYSP comprises 598 nodes and 2791 edges. The network is depicted in **Figure 1A**.

#### 3.1.3 Intersection of active compound targets and disease targets

After classification, disease-related targets were retrieved using the Genecards database. Based on a relevance score greater than 7 from Genecards, duplicates were removed, resulting in 1589 disease-associated targets. Using the online tool Venny 2.1.0 (http://bioinfogp.cnb.csic.es/tools/venny/index.html), these 1589 neuroblastoma-related disease targets were mapped against 461 targets of active compounds from FYSP. This analysis identified 188 common targets, as shown in **Figure 1B**.

**Figure 2:**
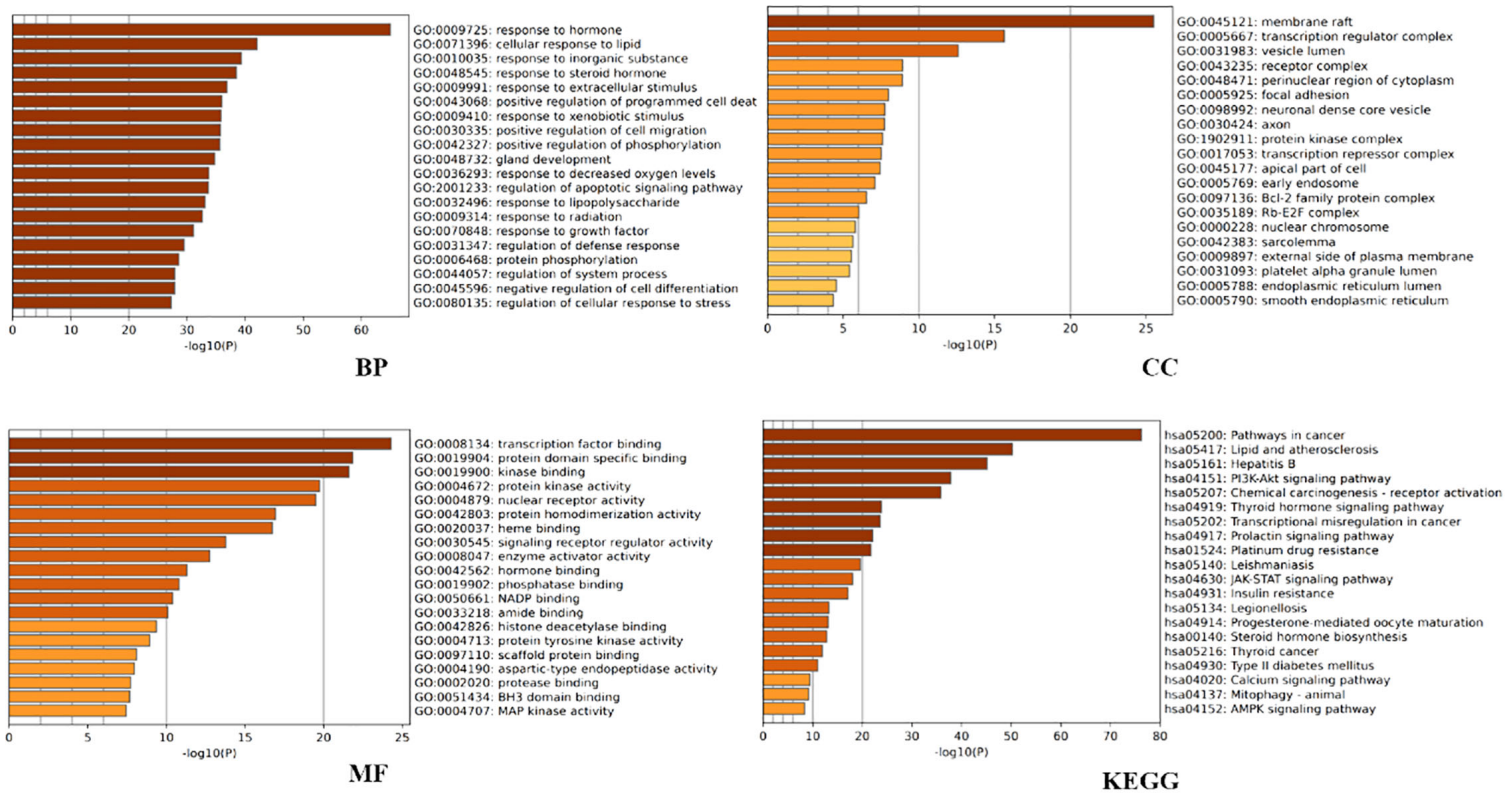
Metascape-based GO and KEGG enrichment analysis of the top 20 pathways.

#### 3.1.4 PPI network analysis and core target selection for FYSP

Disease targets and targets of FYSP were imported into Cytoscape 3.7.2, and a PPI network was constructed using the Bisgenet plugin. The new network consists of 7815 nodes and 177,723 edges. DC, BC, and CC were used to measure the topological importance of target proteins within the network. The higher the DC value of a node, the more crucial that protein is in the PPI network. We constructed a new network containing 2185 nodes and 90,856 edges. Using CytoNCA, DC, BC, and CC were calculated for the 2185 nodes, with a median BC of 486.8085, a CC median of 0.63263, and a DC median of 265, leading to the identification of key target proteins (**Figure 1C**).

**Figure 3:**
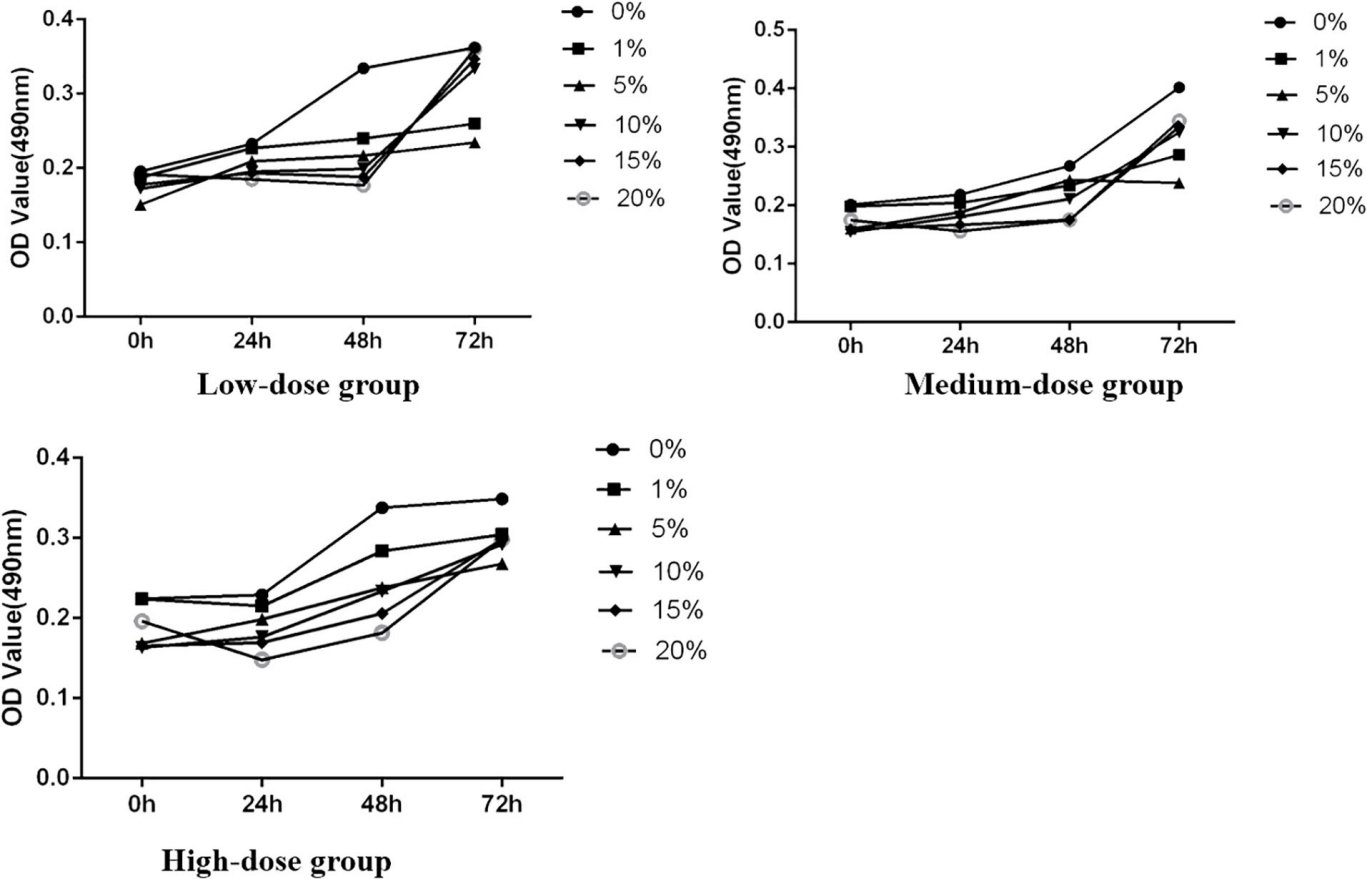
The effect of each concentration on the OD value of SH-SY5Y cells.

#### 3.1.5 GO and KEGG enrichment analysis

Further analysis was conducted to explore the biological effects of FYSP in the treatment of pediatric malignancies. This study conducted GO analysis on 188 intersecting target proteins based on three categories: BP, CC, and MF (14). Within the Metascape database, the top 20 entries were selected for visualization. Biological processes identified include responses to hormones, lipids, inorganic substances, steroid hormones, extracellular stimuli, positive regulation of programmed cell death, responses to external stimuli, positive regulation of cell migration, phosphorylation, gland development, and hypoxia. Molecular functions were enriched in transcription factor binding, protein domain-specific binding, kinase binding, protein kinase activity, nuclear receptor activity, protein homodimerization activity, heme binding, signal receptor regulator activity, enzyme activator activity, and hormone binding (as shown in **Figure 2**).

KEGG pathway analysis was utilized to further elucidate the involvement of target proteins in protein biosynthesis. The analysis revealed that the 188 intersecting target proteins were enriched in 198 pathways (*P* < 0.05). The top 20 pathways, as illustrated in **Figure 2**, included key pathways such as the PI3K/AKT signaling pathway, JAK-STAT signaling pathway, and AMPK signaling pathway.

### 3.2 Experimental validation of FYSP’s inhibition of human neuroblastoma through the PI3K/AKT pathway

#### 3.2.1 Determining optimal experimental conditions

The study initially assessed the inhibitory effect of medicated serum at different concentrations on the proliferation of SH-SY5Y cells over 24, 48, and 72 hours, laying the groundwork for determining subsequent experimental dosing concentrations. The experiments were divided into three dosage levels: low dosage (medicated serum concentrations of 0%, 1%, 5%, 10%, 15%, and 20%), medium dosage (same concentrations), and high dosage (same concentrations). Results indicated that, across these time points, as the concentration of medicated serum increased, there was a corresponding decrease in the viability of SH-SY5Y cells. At the same concentration, cell viability also decreased with prolonged exposure to medicated serum. Statistical analysis revealed that FYSP medicated serum inhibited the growth of SH-SY5Y cells in a time- and dose-dependent manner, with the most significant change in cell proliferation observed at the high dosage of 20% serum concentration. Future experiments will proceed using a high dosage of 20% serum, as shown in **Figure 3**.

#### 3.2.2 siRNA transfection and PI3K expression in SH-SY5Y cells

**Figure 4:**
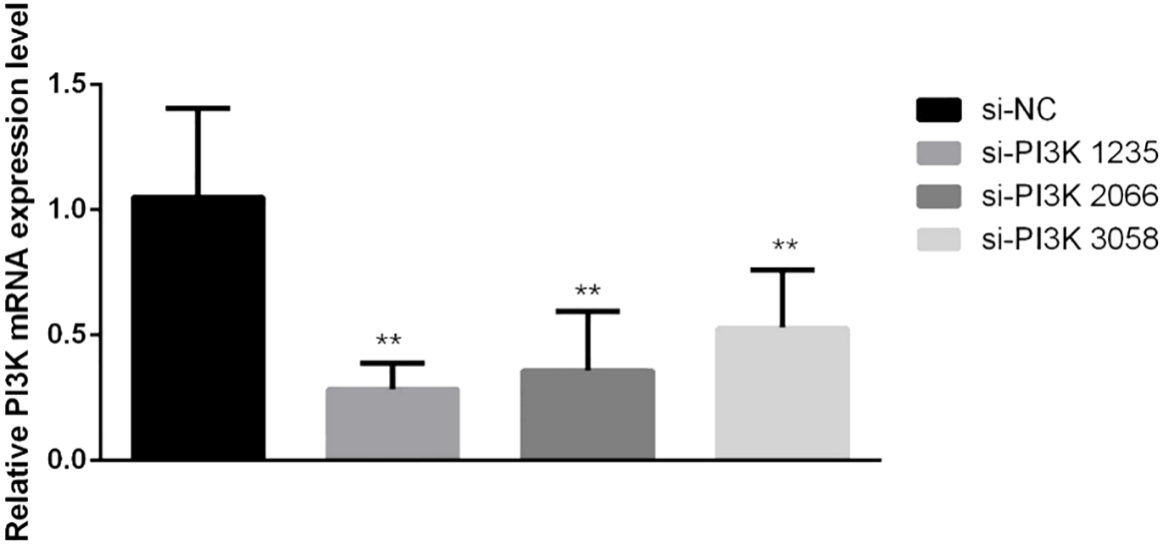
qPCR verification of PI3K siRNA knockdown efficiency after transfection. In the comparisons, significance levels are indicated as *P < 0.05 and **P < 0.01 compared to the si-NC group.

**Figure 5:**
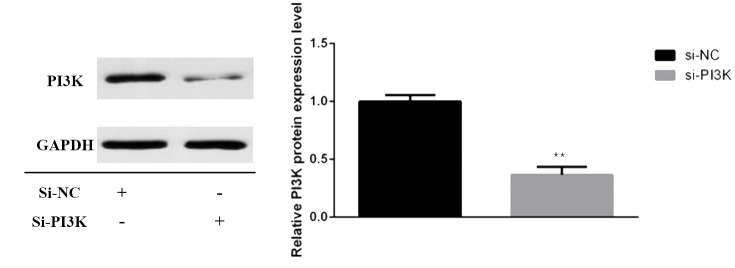
Protein expression of PI3K after transfection. In the comparisons, significance levels are indicated as *P < 0.05 and **P < 0.01 compared to the si-NC group.

siRNAs were transfected into SH-SY5Y cells to knockdown PI3K expression. Forty-eight hours post-transfection, cells were harvested for RNA and protein extraction, followed by quantitative PCR (qPCR) and Western blot analyses to evaluate the knockdown efficiency of the siRNA. The siRNAs used included si-NC (non-specific siRNA control) and three different siRNA sequences targeting PI3K: si-PI3K1235, si-PI3K2066, and si-PI3K3085. qPCR results indicated that all groups experienced a reduction in PI3K mRNA levels, with si-PI3K1235 showing the highest knockdown efficiency (**Figure 4**). In the Western blot assays, the protein expression in the si-PI3K groups was significantly lower than that in the si-NC group, with statistically significant differences (**Figure 5**). Through the assessment of RNA and protein levels, it was confirmed that the siRNA sequence si-PI3K1235 was the most effective at reducing PI3K expression. Subsequent experiments will utilize the si-PI3K1235 sequence.

#### 3.2.3 Effects of FYSP on cell proliferation

**Figure 6:**
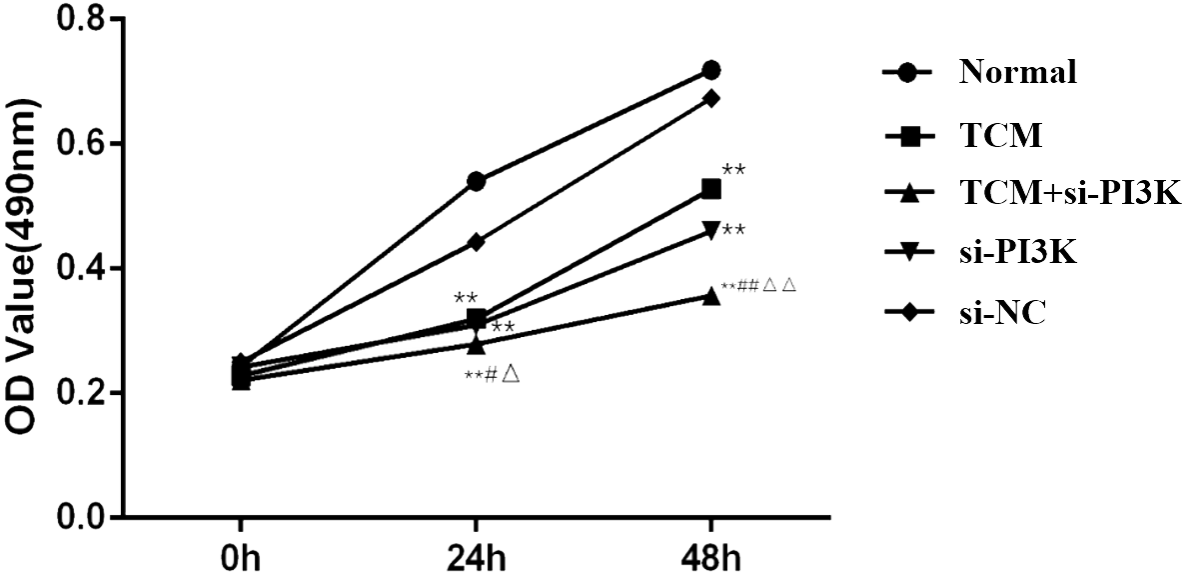
Effects on OD values of SH-SY5Y cells across different groups. Compared to the normal control group: *P < 0.05, **P < 0.01; compared to the TCM group: #*P* < 0.05, ##*P* < 0.05; compared to the si-PI3K group: △*P* < 0.05, △△*P* < 0.01.

Cell proliferation assays were conducted to explore the effects of FYSP on SH-SY5Y cell proliferation, as shown in **Figure 6**. Results indicated that after 24 and 48 hours of intervention, the OD values of the TCM group, the si-PI3K + TCM group, and the si-PI3K group were all significantly lower than those of the normal control group (*P* < 0.01). At both 24 and 48 hours, the OD values for the si-PI3K + TCM group were lower than those of the TCM group and the si-PI3K group, with statistically significant differences (*P* < 0.05 or *P* < 0.01). After 48 hours, there was no statistically significant difference between the negative control group and the normal control group (*P* > 0.05).

The OD values were converted into growth inhibition rates, revealing the impact of FYSP on the growth inhibition of SH-SY5Y cells, as detailed in **Figure 3**. At 24 and 48 hours, the inhibition rates in the TCM group, si-PI3K + TCM group, and si-PI3K group were all significantly higher than those in the normal control group (*P* < 0.01). At 24 hours, there was no statistical difference in inhibition rates between the si-PI3K + TCM group and the TCM group (*P* > 0.05); however, at 48 hours, the inhibition rate in the si-PI3K + TCM group was higher than those in the TCM group and the si-PI3K group, with significant differences (*P* < 0.05 or *P* < 0.01). These results demonstrate that the medicated serum of FYSP significantly inhibits the proliferation of SH-SY5Y cells.

#### 3.2.4 Effects of FYSP on the cell cycle

**Figure 7:**
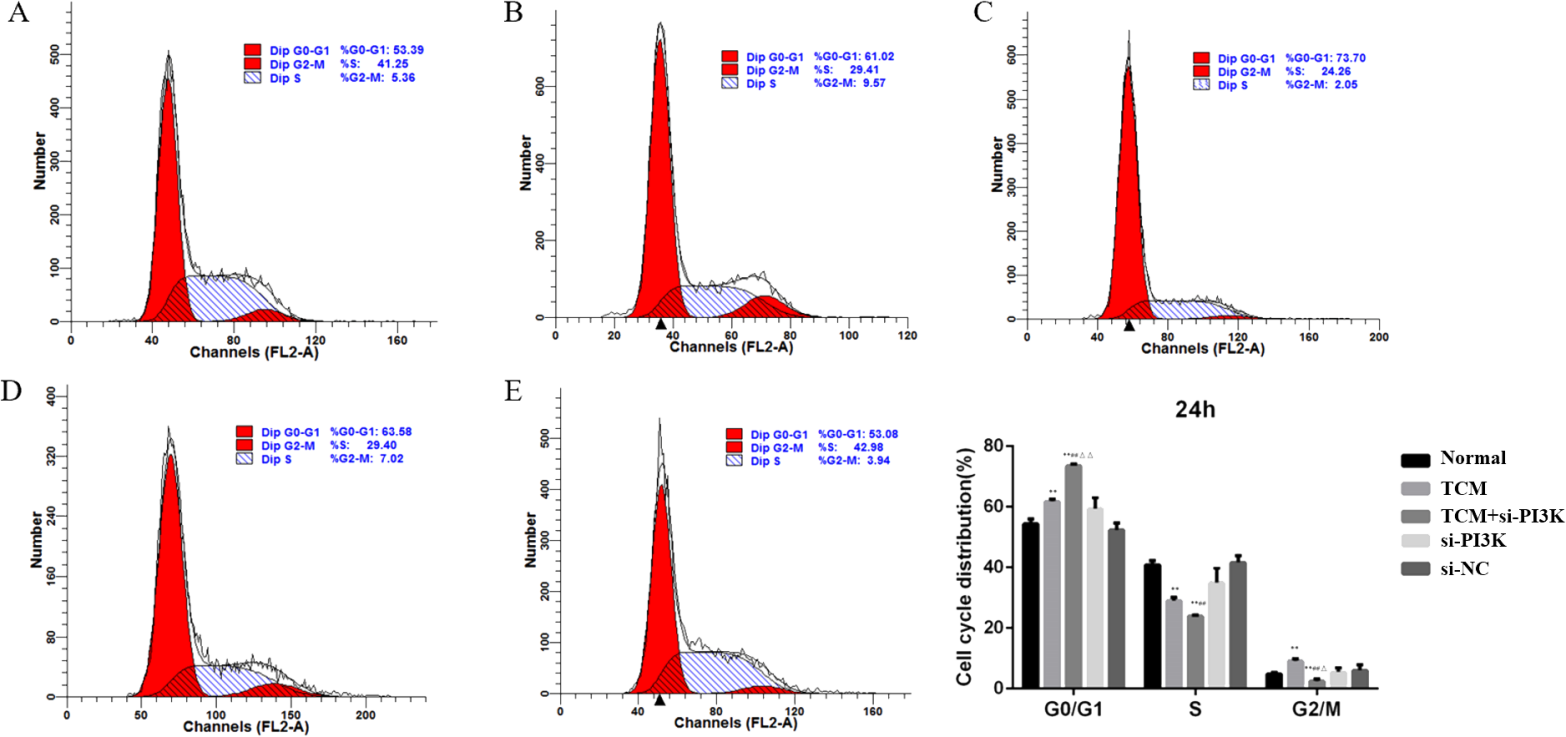
The influence of each group on SH-SY5Y cell cycle detected by flow cytometry. Labels **(A-E)** represent the normal control group, TCM group, si-PI3K + TCM group, si-PI3K group, and negative control group in flow cytometry cell cycle diagrams; compared to the normal control group *P < 0.05, **P < 0.01; compared to the TCM group #*P* < 0.05, ##*P* < 0.01; compared to the si-PI3K group △*P* < 0.05, △△*P* < 0.01.

Cell cycle analysis was performed using flow cytometry to assess the impact of FYSP on the SH-SY5Y cell cycle, as shown in **Figure 7**. Cell proliferation assays indicated significant cellular inhibition as early as 24 hours post-intervention, prompting cell cycle analysis at this time point. The results revealed an increased proportion of cells arrested in the G0/G1 phase in the TCM group, si-PI3K + TCM group, and si-PI3K group compared to the normal control group, with the TCM group and si-PI3K + TCM group showing significant differences (*P* < 0.01). The proportion of cells in the G0/G1 phase was higher in the si-PI3K + TCM group compared to both the TCM group and the si-PI3K group, with significant differences (*P* < 0.01). There was no significant difference between the normal control group and the negative control group (*P* > 0.05).

#### 3.2.5 Effects of FYSP on cell migration

**Figure 8:**
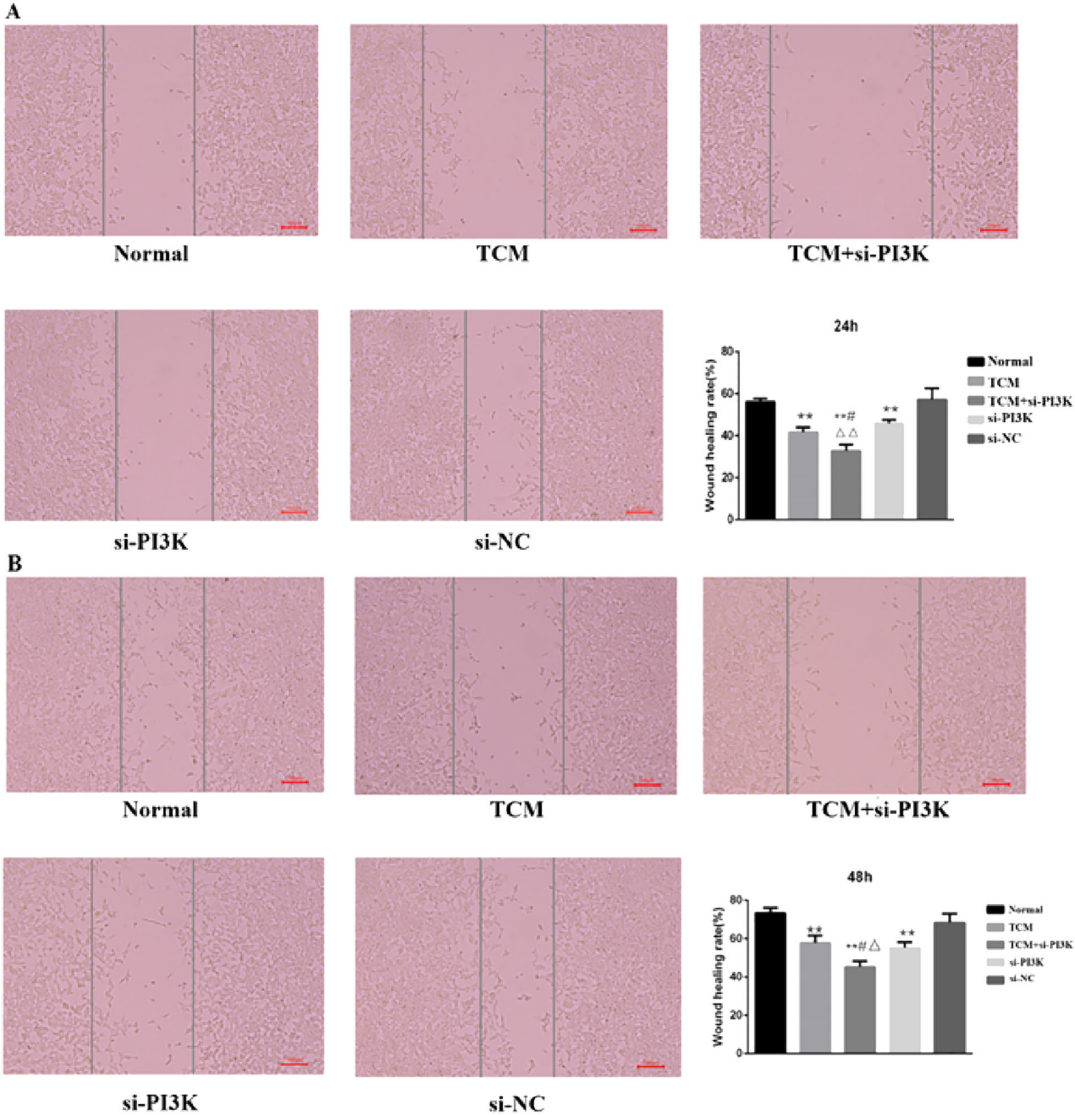
The cell migration diagram and the migration rate of each group in the scratch test. **(A)** 24 hours; **(B)** 48 hours. Compared to the normal control group *P < 0.05, **P < 0.01; compared to the TCM group #*P* < 0.05, ##*P* < 0.05; compared to the si-PI3K group △*P* < 0.05, △△*P* < 0.01.

Microscopic Observation: 24 hours after treatment, the scratch distances in both the normal control group and the negative control group had noticeably narrowed as cells gradually migrated toward the central blank area. By 48 hours, the narrowing of the scratch width was more pronounced, and migrating cells almost covered the original distance, with the scratches nearing closure. The migration distances of the groups after 48 hours of intervention were as follows: si-PI3K + TCM group < si-PI3K group < TCM group < negative control group < normal control group. The si-PI3K + TCM group, si-PI3K group, and TCM group showed a greater scratch width and lower cell density compared to the normal and negative control groups, as shown in **Figure 8A, B**.

Scratch Assay Results: Migration rates obtained from the scratch assay were compared among the groups. Post-intervention, at the same time points, the migration rates of the TCM group, si-PI3K + TCM group, and si-PI3K group were all lower than that of the normal control group, with significant differences (*P* < 0.01). At 24 and 48 hours, the migration rate of the si-PI3K + TCM group was lower than those of the TCM and si-PI3K groups, with statistically significant differences (*P* < 0.05 or *P* < 0.01). No significant statistical differences were observed between the normal control group and the negative control group at 24 and 48 hours, as depicted in **Figure 8A, B**.

#### 3.2.6 Effects of FYSP on cell apoptosis

**Figure 9:**
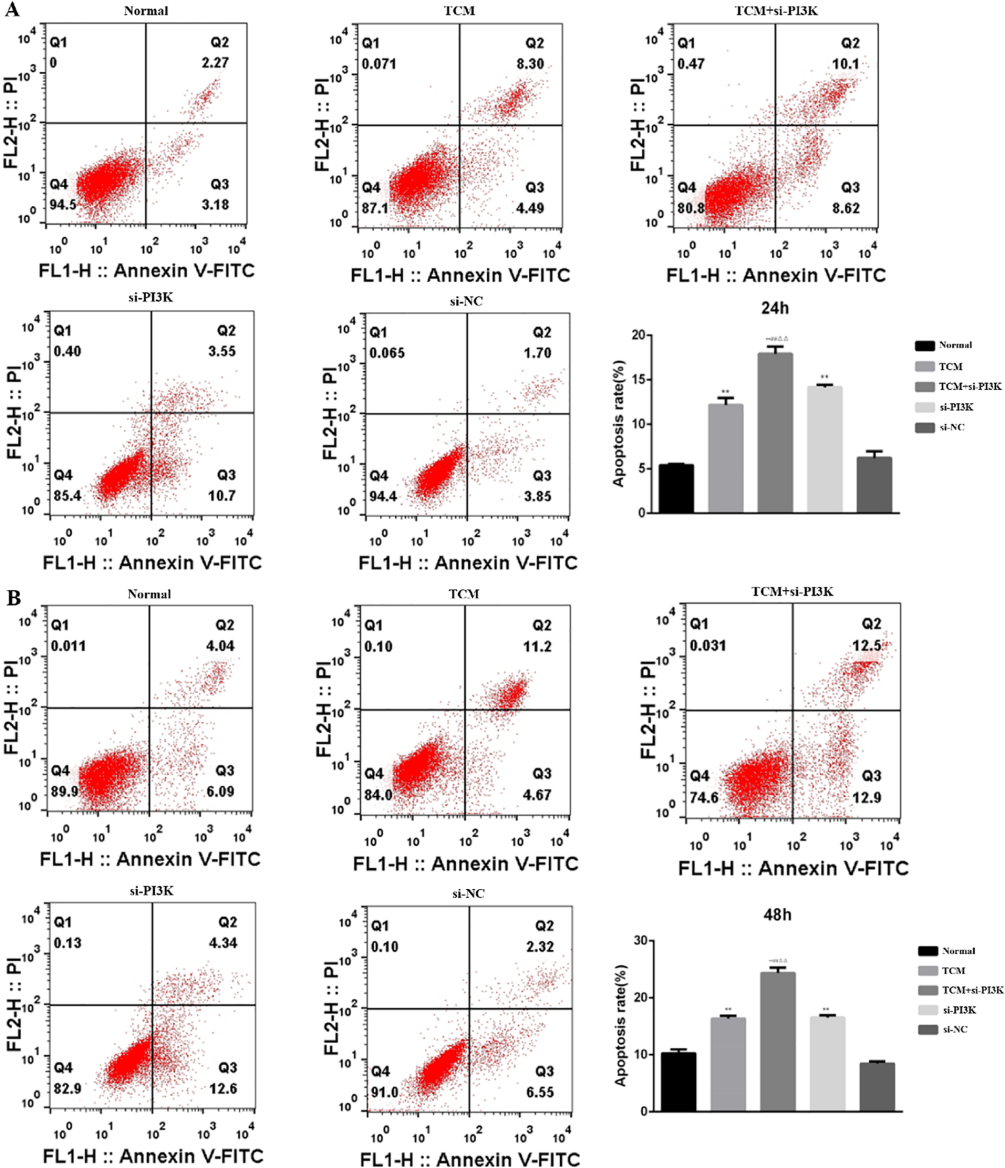
The effect of each group on the apoptosis of SH-SY5Y cells detected by flow cytometry. **(A)** 24 hours; **(B)** 48 hours. Compared to the normal control group *P < 0.05, **P < 0.01; compared to the TCM group #*P* < 0.05, ##*P* < 0.01; compared to the si-PI3K group △*P* < 0.05, △△*P* < 0.01.

Detection of Cell Apoptosis: Cell apoptosis was assessed using flow cytometry to evaluate the impact of FYSP on apoptosis in neuroblastoma SH-SY5Y cells, as shown in **Figure 9**. The results indicate that after 24 and 48 hours of intervention, the apoptosis rates in the TCM group, si-PI3K + TCM group, and si-PI3K group were higher than in the normal control group, with statistically significant differences (*P* < 0.01). The apoptosis rate in the si-PI3K + TCM group was higher than those in the TCM and si-PI3K groups (*P* < 0.01). No significant statistical differences were observed between the normal control group and the negative control group (*P* > 0.05). Compared to the normal control group, both early and late apoptosis induced by the TCM group and the si-PI3K + TCM group showed statistically significant differences (*P* < 0.05 or *P* < 0.01). These findings suggest that the medicated serum of FYSP can induce apoptosis in SH-SY5Y cells.

**Figure 10:**
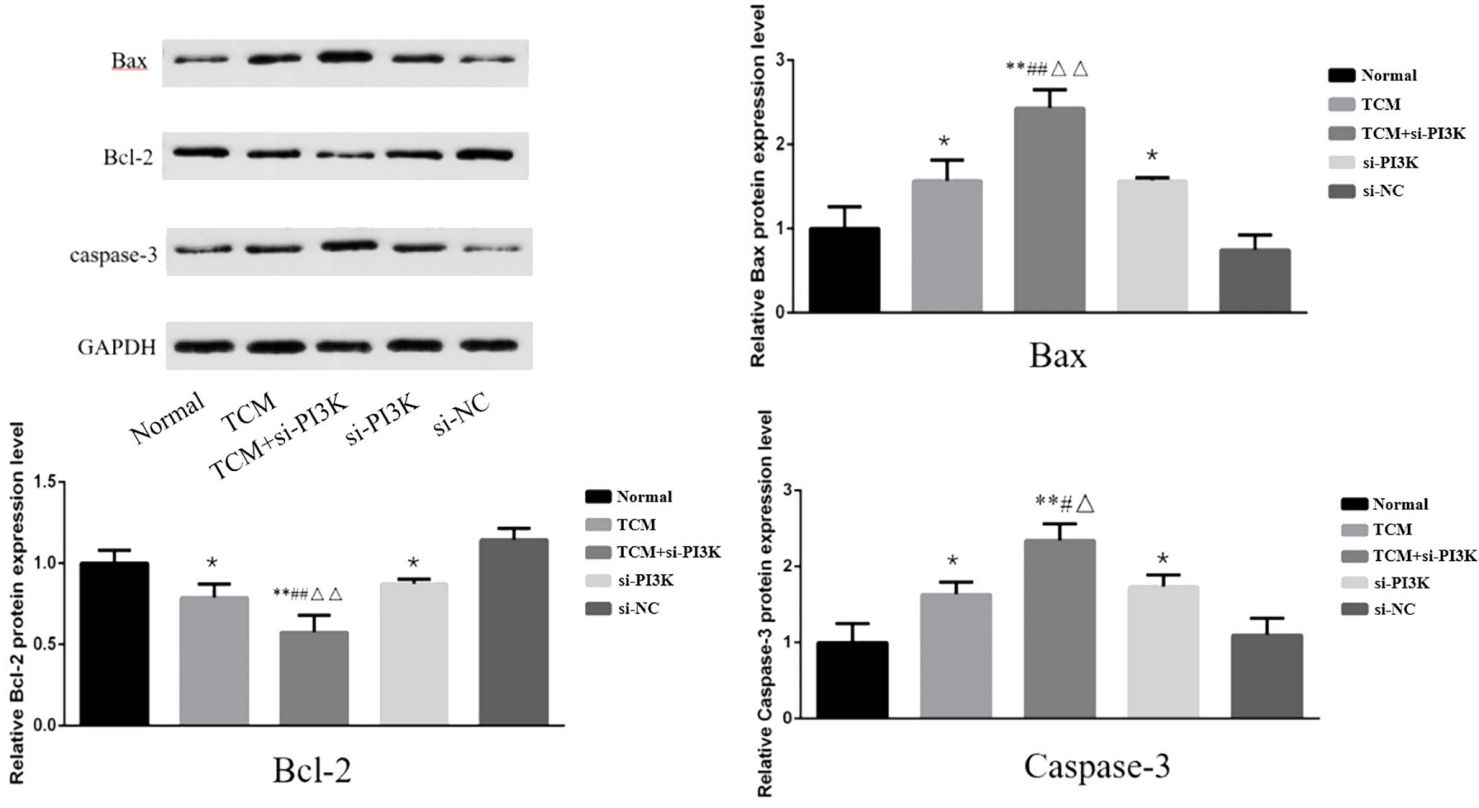
The effect of each group on the expression of apoptosis-related proteins in SH-SY5Y cells. Comparison with the normal control group is indicated by *P < 0.05, **P < 0.01; comparison with the TCM group is marked by #*P* < 0.05, ##*P* < 0.01; comparison with the si-PI3K group is denoted by △*P* < 0.05, △△*P* < 0.01.

Expression of Apoptosis-Related Proteins: To further validate the apoptotic regulatory effects after treating SH-SY5Y cells with FYSP, Western blot analysis was used to measure levels of apoptosis-related proteins. Bar graphs based on densitometric values were constructed to visualize the expression levels of apoptotic proteins, as shown in **Figure 10**. The results indicate that compared to the normal control group, the TCM group, si-PI3K + TCM group, and si-PI3K group exhibited increased expression levels of pro-apoptotic genes Bax and Caspase-3 and decreased expression levels of the anti-apoptotic gene Bcl-2, with statistically significant differences (*P* < 0.05 or *P* < 0.01). Expression levels of Bax and Caspase-3 were higher in the si-PI3K + TCM group than in the TCM and si-PI3K groups, while Bcl-2 levels were lower (*P* < 0.05 or *P* < 0.01).

#### 3.2.7 Effects of FYSP on the expression of proteins related to the PI3K/AKT signaling pathway

**Figure 11:**
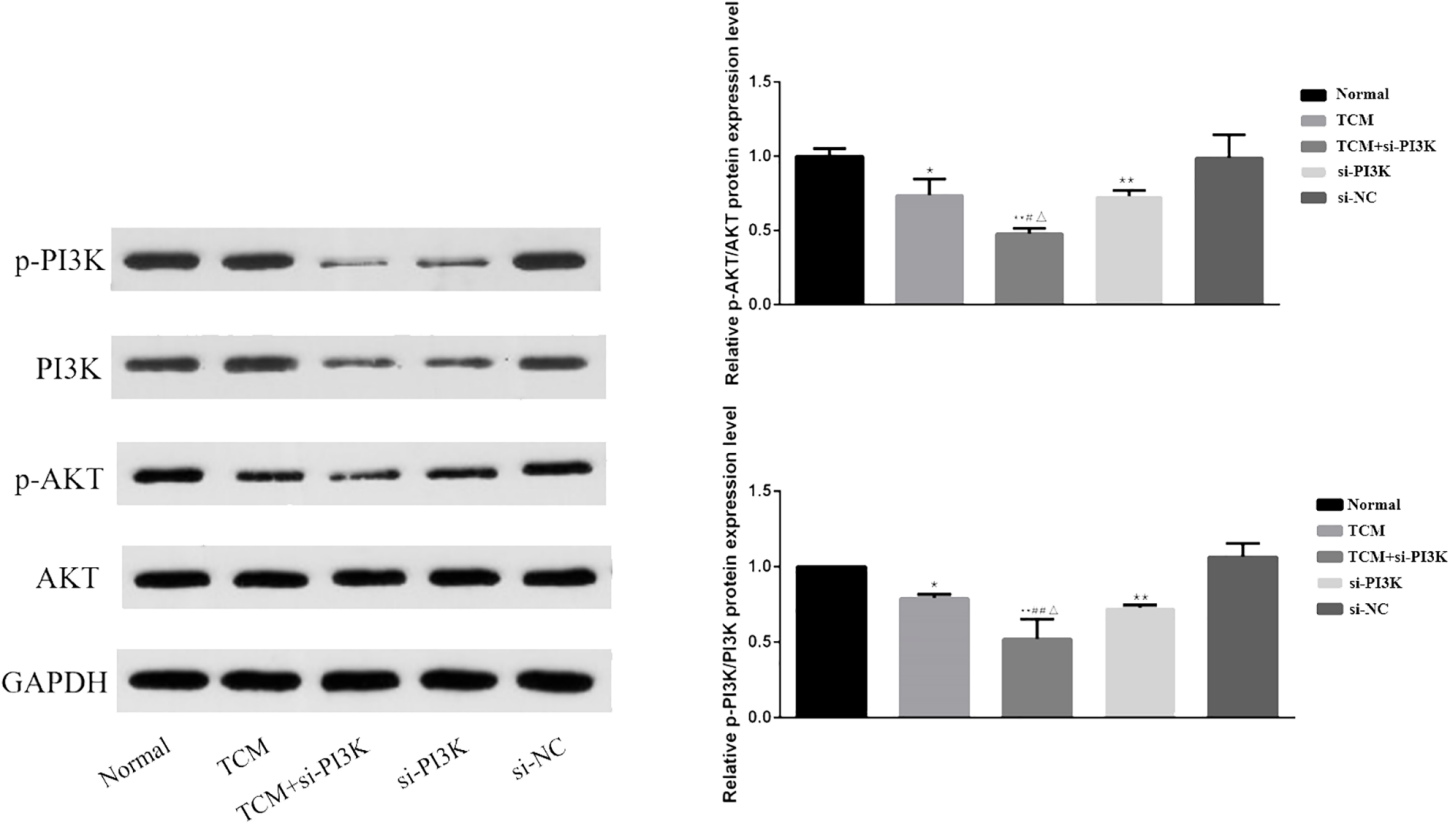
Effect of each group on the expression of proteins related to PI3K/AKT signaling pathway. Comparison with the normal control group is indicated by *P < 0.05, **P < 0.01; comparison with the TCM group is marked by #*P* < 0.05, ##*P* < 0.01; comparison with the si-PI3K group is denoted by △*P* < 0.05, △△*P* < 0.01.

Western blot analysis was used to measure the expression of proteins associated with the PI3K/AKT signaling pathway in SH-SY5Y cells. Bar graphs based on densitometric values were constructed to illustrate the levels of pathway-related protein expression, as shown in **Figure 11**. The results indicate that there was no significant difference in the expression of AKT between the groups (*P* > 0.05). However, the expression of phosphorylated AKT was significantly reduced in the TCM group, si-PI3K + TCM group, and si-PI3K group compared to the normal control group, with statistically significant differences (*P* < 0.05 or *P* < 0.01). The expression of phosphorylated AKT in the si-PI3K + TCM group was lower than in the TCM and si-PI3K groups (*P* < 0.05). The expression of PI3K was reduced in the si-PI3K + TCM group and si-PI3K group. Phosphorylated PI3K expression was significantly reduced in the TCM group, si-PI3K + TCM group, and si-PI3K group compared to the normal control group, with statistically significant differences (*P* < 0.05 or *P* < 0.01); the expression of phosphorylated PI3K in the si-PI3K + TCM group was lower than in the TCM and si-PI3K groups (*P* < 0.05 or *P* < 0.01).

## 4 Discussion

Neuroblastoma occupies a significant position among pediatric malignancies, with its complex biological characteristics and diverse treatment responses demanding more effective therapeutic strategies (6). TCM offers the advantages of multiple components and multiple targets, allowing comprehensive modulation of various mechanisms involved in the development of neuroblastoma, fully embodying the holistic concept of TCM. This study systematically and thoroughly analyzed the mechanism of action of FYSP in neuroblastoma using network pharmacology combined with *in vitro* experimental validation specifically in SH-SY5Y cells. Our findings demonstrate that FYSP can promote apoptosis in SH-SY5Y neuroblastoma cells by modulating the PI3K-Akt signaling pathway, thereby exerting a protective effect.

The SH-SY5Y cell line is frequently used in neuroblastoma research due to its ability to retain neuroblastoma characteristics, such as specific neuronal markers and enzyme activities. Its use in this study allows for a focused analysis of neuroblastoma cellular mechanisms and drug interactions, providing insights that are relevant to the biological behaviors observed in neuroblastoma. Our research revealed that FYSP can induce cell cycle arrest at the G0/G1 phase in SH-SY5Y cells. This effect was further enhanced when FYSP was used alone or in combination with a recombinant plasmid targeting the PI3K gene in SH-SY5Y cells. Through assays measuring cell proliferation, cell cycle, apoptosis, and cell migration, results indicated that FYSP could inhibit SH-SY5Y cell proliferation and migration, induce apoptosis, and block the cell cycle at the G0/G1 phase. The effects were amplified when TCM and si-PI3K were used in conjunction; Western blot experiments revealed downregulated expression levels of p-PI3K and p-AKT, further enhanced by the combination with si-PI3K. Therefore, we hypothesize that FYSP may inhibit the activity of the PI3K/AKT signaling pathway, arresting SH-SY5Y cells at the G0/G1 phase, inhibiting cell proliferation and migration, and inducing apoptosis.

Avoidance of apoptosis, an intrinsic cellular death program, is a hallmark of human cancers, including neuroblastoma (15). Additionally, the cytotoxic activities of common anticancer treatments such as chemotherapy, γ radiation, or immunotherapy, are primarily mediated by inducing tumor cell apoptosis (16). Thus, better understanding the signaling pathways and molecules regulating apoptosis in neuroblastoma cells could pave new avenues for designing molecular targeted therapies for neuroblastoma (17). Apoptotic cells exhibit characteristic features such as cytoplasmic shrinkage, phosphatidylserine (PS) membrane exposure, chromatin condensation, and DNA fragmentation (18). Apoptosis typically occurs along two principal pathways: the extrinsic pathway activated by death receptors and the intrinsic pathway mediated by mitochondria. It is regulated by a balance between pro-apoptotic and anti-apoptotic proteins of the Bcl-2 family, initiator caspases (such as caspase-8 and -9), and effector caspases (such as caspase-3 and -7) (19, 20). These events are hallmarks of apoptosis and are commonly used to identify pathways involved in cell death. Our study has found that FYSP can effectively promote apoptosis in SH-SY5Y cells.

Enrichment analysis suggests that numerous signaling pathways might be involved in the treatment of neuroblastoma by FYSP. The PI3K-Akt signaling pathway is not only the most crucial pathway associated with neuroblastoma but also contains key targets in the PPI network. Thus, we targeted the PI3K-Akt signaling pathway for validation. The PI3K-Akt signaling pathway is closely linked with the progression of many cancers. During tumor progression, the PI3K-Akt pathway can be activated by various types of cellular stimuli or toxic injuries, regulating transcription, translation, proliferation, growth, and survival (7, 21). The PI3K-Akt pathway has been found to exert a protective effect in neuroblastoma by promoting apoptosis (22, 23). Growth factors binding to their RTKs stimulate class Ia PI3Ks, while chemokines, hormones, and neurotransmitters binding to G protein-coupled receptors (GPCRs) stimulate class Ib PI3Ks (24). PI3K catalyzes the production of phosphatidylinositol-3,4,5-trisphosphate (PIP3) in the cell membrane. PIP3 acts as a second messenger, helping to activate Akt (25). Akt can regulate many key cellular processes, such as apoptosis, protein synthesis, metabolism, and the cell cycle, by phosphorylating its substrates, inhibiting the growth and survival of cancer cells (26, 27). The PI3K/AKT pathway is an upstream pathway of the anti-apoptotic protein Bcl-2; inhibition of this pathway can lead to cell apoptosis by reducing Bcl-2 expression (28). In our study, FYSP was shown to inhibit the PI3K/AKT pathway, confirmed by adding siRNA targeting PI3K (si-PI3K). These findings suggest that the apoptosis induced by FYSP in SH-SY5Y cells is regulated by the PI3K/AKT pathway.

## 5 Limitations

While this study provides significant insights into the effects of FYSP on SH-SY5Y neuroblastoma cells, there are notable limitations that must be considered when interpreting the results. Firstly, the effects of FYSP were exclusively examined in SH-SY5Y neuroblastoma cells. This focus limits the generalizability of the findings to other cell types, particularly non-cancerous cells. The potential cytotoxic effects of FYSP on normal, healthy cells were not assessed in this study. Understanding whether FYSP selectively targets cancer cells without harming normal cells is crucial for evaluating its safety and therapeutic potential. Future studies should aim to investigate the impact of FYSP on a range of non-cancerous cells to establish a comprehensive safety profile. Additionally, our findings are derived solely from *in vitro* experiments, which provide limited information about the complex interactions and pharmacokinetics that occur in a living organism. These limitations underscore the need for further research to fully elucidate the therapeutic potential of FYSP and ensure its efficacy and safety in broader clinical applications.

## 6 Conclusion

In summary, our study provides a detailed analysis of the active components of FYSP and their impact on molecular pathways in SH-SY5Y neuroblastoma cells. We demonstrated that FYSP induces cell cycle arrest and apoptosis through modulation of the PI3K/Akt signaling pathway. These findings underscore the potential mechanistic basis for FYSP’s effects *in vitro*.

## Data Availability

The original contributions presented in the study are included in the article/supplementary material. Further inquiries can be directed to the corresponding author.
